# Soluble ST2 in the prediction of heart failure and death in patients with atrial fibrillation

**DOI:** 10.1002/clc.23799

**Published:** 2022-02-21

**Authors:** Rungroj Krittayaphong, Satchana Pumprueg, Poom Sairat

**Affiliations:** ^1^ Division of Cardiology, Department of Medicine, Faculty of Medicine Siriraj Hospital Mahidol University Bangkok Thailand

**Keywords:** history of heart failure, nonvalvular atrial fibrillation, patients, prognostic significance, soluble ST2 level

## Abstract

**Background:**

Biomarkers may be a useful marker for predicting heart failure (HF) or death in patients with atrial fibrillation (AF).

**Hypothesis:**

Soluble ST2 (sST2) may be a good biomarker for the prediction of HF or death in patients with AF.

**Methods:**

This is a prospective study of patients with nonvalvular AF. Clinical outcomes were HF or death. Clinical and laboratory data were compared between those with and without clinical outcomes. Univariate and multivariate analysis was performed to determine whether sST2 is an independent predictor for heart failure or death in patients with nonvalvular AF.

**Results:**

A total of 185 patients (mean age: 68.9 ± 11.0 years) were included, 116 (62.7%) were male. The average sST2 and N‐terminal pro‐brain natriuretic peptide (NT‐proBNP) levels were 31.3 ± 19.7 ng/ml and 2399.5 ± 6853.0 pg/ml, respectively. Best receiver operating characteristic (ROC) cut off of sST2 for predicting HF or death was 30.14 ng/ml. Seventy‐three (39.5%) patients had an sST2 level ≥30.14 ng/ml, and 112 (60.5%) had an sST2 level <30.14 ng/dl. The average follow‐up was 33.1 ± 6.6 months. Twenty‐nine (15.7%) patients died, and 33 (17.8%) developed HF during follow‐up. Multivariate analysis revealed that high sST2 to be an independent risk factor for death or HF with a HR and 95% CI of 2.60 (1.41–4.78). The predictive value of sST2 is better than NT‐proBNP, and it remained significant in AF patients irrespective of history of HF, and NT‐proBNP levels.

**Conclusions:**

sST2 is an independent predictor of death or HF in patients with AF irrespective of history of HF or NT‐proBNP levels.

## 1 INTRODUCTION

Non‐valvular atrial fibrillation (AF) is one of the most common cardiac arrhythmias,^1^ and the prevalence of AF increases in older adult population.^2^ Heart failure (HF) is one of the coexisting conditions frequently seen in patients with AF,^3^, ^4^ and the prevalence of HF also increases in older adults.^5^ When AF and HF coexist in a patient, it is often difficult to determine which condition is the cause, and which is the effect.^6^ Practice guidelines mainly focus on stroke prevention in patients with AF and HF is often overlooked. Results from the Global Anticoagulant Registry in the Field‐Atrial Fibrillation (GARFIELD‐AF) registry, which is a large global registry of patients with newly diagnosed AF, revealed a rate of HF of 2.41 per 100 person‐years, which is greater than the rate of ischemic stroke, major bleeding, and cardiovascular death.^7^ Recent European Society of Cardiology (ESC) guideline for management of AF emphasizes the treatment of comorbidities, such as hypertension, diabetes, and HF.^8^

Natriuretic peptide, such as brain natriuretic peptide (BNP) and N‐terminal pro‐BNP (NT‐proBNP), has been shown to be both a diagnostic and prognostic biomarker for HF.^9^ Soluble ST2 (sST2) is another biomarker that has been demonstrated to be a good prognostic marker in patients with HF.^10^, ^11^ American College of Cardiology (ACC) guideline for management of patients with HF suggests that sST2 may be useful as an additive biomarker for prognosis of patients with HF.^12^ The objectives of this study were to determine (1) the prognostic value of sST2 for HF and death in patients with AF; (2) the prognostic value of sST2 for HF and death in patients with AF with and without history of HF; and (3) whether the prognostic value of sST2 for HF and death in patients with AF is independent of NT‐proBNP level.

## 2 METHODS

### 2.1 Study population

This is a prospective study. Patients who were at least 18 years of age with a diagnosis of nonvalvular AF were prospectively enrolled. The presence of AF was confirmed by 12‐lead electrocardiography (ECG) or ambulatory ECG monitoring. Patients with at least one of the following criteria were excluded: (1) rheumatic mitral stenosis; (2) mechanical heart valve; (3) AF from transient reversible cause, such as pneumonia; (4) pregnancy; (5) life expectancy less than 3 years; (6) unwilling to participate; (7) hospitalization within 1 month before study enrollment; (8) ongoing participation in a clinical trial; and/or (9) inability to attend follow‐up appointments. The protocol for this study was approved by the Institutional Review Board (IRB) of the Faculty of Medicine Siriraj Hospital, Mahidol University, Bangkok, Thailand, and all patients gave written informed consent to participate.

### 2.2 Study protocol and data collection

Baseline data were collected and recorded from medical record reviews and patient interviews. Included patients were followed‐up every 6 months for 3 years. In addition to study‐related data that were collected at each follow‐up visit, the authors investigated for, determined, and recorded the occurrence of study outcomes (HR or death) that occurred during the preceding six months.

The following data were collected: demographic data; type, duration, and symptom of AF; left ventricular ejection fraction (LVEF) from echocardiogram; comorbid conditions, including history of HF, coronary artery disease (CAD), ischemic stroke/transient ischemic attack (TIA), diabetes mellitus (DM), hypertension (HT), dyslipidemia (DLP), smoking, implantable devices, and dementia; medications; and, laboratory data, such as creatinine clearance for the calculation for chronic kidney disease (CKD) and renal replacement therapy (RRT), hematocrit for assessment of anemia, NT‐proBNP, and sST2.

### 2.3 Definitions

CAD was defined as the presence of significant stenosis of at least one major coronary artery by coronary angiogram or coronary computed tomography (CT) angiography, or history of documented myocardial infarction or coronary revascularization or positive stress imaging either by nuclear stress test, magnetic resonance imaging, or echocardiography. Anemia was defined as hemoglobin level <13 g/dl for males, and <12 g/dl for females. CKD in this study was defined as CKD stages 3–5 or an estimated glomerular filtration rate (eGFR [ml/min/1.73 m^2^]) by Chronic Kidney Disease Epidemiology Collaboration (CKD‐EPI) formula less than 60 ml/min.

### 2.4 Laboratory investigations

sST2 was measured from plasma samples using a high‐sensitivity sandwich monoclonal immunoassay (Presage ST2 Assay, Critical Diagnostics). The sST2 assay had a within‐run coefficient of less than 2.5%, a total coefficient of variation of 4%, and a limit of detection of 1.31 ng/ml. NT‐proBNP was measured from plasma using a commercially available immunoassay (Elecsys NT‐proBNP assay, Roche Diagnostics). eGFR (ml/min/1.73 m^2^) was calculated using the CKD‐EPI formula.

### 2.5 Outcomes

The primary outcomes of this study were HF or death. We used standard definition for cardiovascular endpoint events proposed by the American College of Cardiology (ACC) and American Heart Association (AHA).^13^ HF was defined an urgent, unscheduled clinic or emergency department visit or hospital admission, with a primary diagnosis of HF, where the patient exhibits new or worsening symptoms of HF on presentation, has objective evidence of new or worsening HF, and receives initiation or intensification of treatment specifically for HF. Objective evidence consists of at least two physical examination findings OR at least one physical examination finding and at least one laboratory criterion of new or worsening HF on presentation. Death was subcategorized into cardiovascular (CV) death, non‐CV death, or undetermined cause. To minimize the bias, all outcomes were confirmed by a separate adjudication team. The sample size of this registry was enough to determine the differences in outcome between two groups with 90% power.

### 2.6 Statistical analysis

Continuous data were compared by the Student's *t*‐test for unpaired data, and are described as mean ± standard deviation (SD). Categorical data were compared by *χ* ^2^ test or Fisher's exact test, and are described as number and percentage. Clinical outcome data are shown as proportion of outcome in each group, and rate of outcome per 100 person‐years with 95% confidence interval (CI). Kaplan‐Meier estimate was performed to assess the time‐to‐event as the probability of surviving divided by the number of patients at risk. Log‐rank test was performed to compare the difference in survival probability between groups. Univariate and multivariate analysis was performed using Cox proportional hazard function to assess the effect of baseline variables on clinical outcomes. The results are presented as hazard ratio and 95% confidence interval. The primary analysis was based on the sST2 cut‐off derived from receiver operating characteristics (ROC) curve analysis. Sensitivity analysis was performed (1) by using median of sST2 as a cut off (2) by comparing four groups of sST2 separated by quartiles (3) by treating sST2 as continuous data and testing the effect of sST2 on heart failure or death, death, and heart failure outcome by cubic spline graph. A *p*‐value of <.05 was considered statistically significant, and SPSS Statistics software (SPSS, Inc.) and R version 3.6.3 was used to perform data analyses.

## 3 RESULTS

### 3.1 Baseline characteristics

Of the 185 patients that were enrolled, 116 (62.7%) were male. The average age of patients was 68.9 ± 11.0 years, and the average sST2 level was 31.3 ± 19.7 ng/ml (median and interquartile range [IQR]: 26.78 and 18.54–38.38 ng/ml). The average NT‐proBNP level was 2399 ± 6853 pg/ml (median and IQR: 974.4 and 490.9–1841.0 pg/ml).

### 3.2 Clinical outcomes

**Figure 1:**
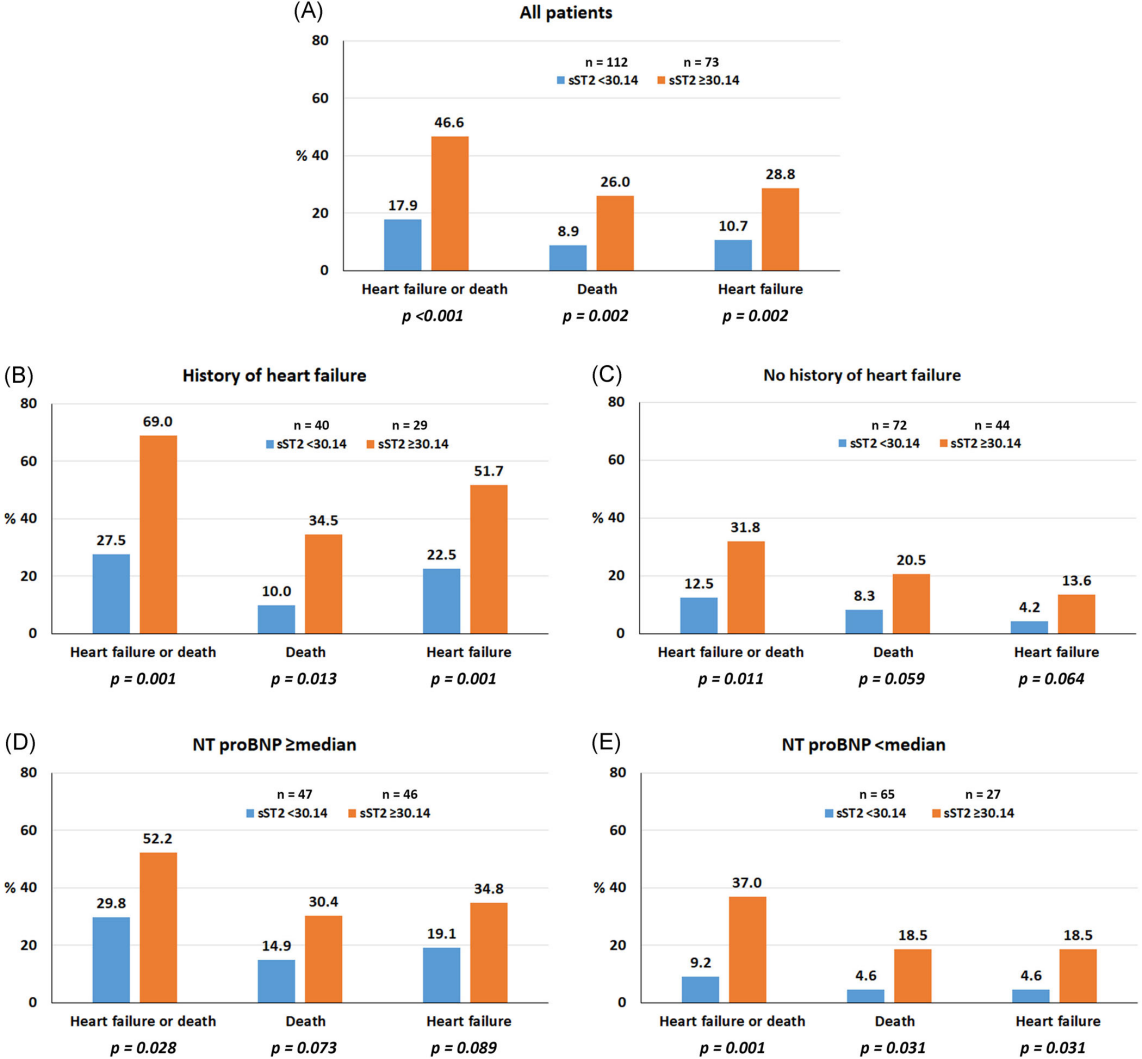
Rate of heart failure (HF) and death according to soluble sST2 group for (A) all patients, (B) patients with history of HF, (C) patients no history of HF, (D) patients with N‐terminal pro‐brain natriuretic peptide (NT‐proBNP) level ≥median, and (E) patients with NT‐proBNP level <median

The average follow‐up duration was 33.1 ± 6.6 months or 502.2 persons‐year. There were 54 patients with death or heart failure during follow‐up (29 deaths and 33 heart failures). Baseline characteristics of patients with and without clinical outcome are shown in Table 1. Older age, history of HF, CAD, DM, HT, CKD, RRT, anemia, low LVEF, and elevated sST2 level were all found to be significantly associated with an increased risk of HF or death. From ROC analysis the best cut‐off of sST2 for death or heart failure was 30.14 ng/ml (area under the curve of 0.69). Seventy‐three (39.5%) patients had an sST2 level ≥30.14 ng/ml, and 112 (60.5%) had an sST2 level <30.14 ng/dl. The proportion of patients with clinical outcomes compared between patients with sST2 level <30.14 and sST2 level ≥30.14 ng/ml is shown in Figure 1. Sixty‐nine (37.3%) patients in our study had history of HF. The differences between the 2 sST2 groups are also shown in patients with and without history of HF, and in patients with NT‐proBNP <median and ≥median. The median (IQR) rate of HF or death, death, and HF was 10.75 (8.08–14.03), 5.77 (3.87–8.29), 6.57 (4.52–9.23) per 100 persons‐years, respectively. The incidence rate of clinical outcomes in patients with sST2 < 30.14 ng/ml and ≥30.14 ng/ml is shown in Table S1. The incidence rate of clinical outcomes was increased in patients with sST2 ≥ 30.14 ng/ml. Table S1 also demonstrated a higher incidence rate of each outcome for patients with sST2 ≥ 30.14 ng/ml regardless of history of heart failure and NT‐proBNP levels.

**Table 1:** Baseline characteristics of NVAF patients compared between those with and without HF or death

Variables	All (*n* = 185)	HF or death (*n* = 54)	No HF or death (*n* = 131)	*p*
Age (years)	68.9 ± 11.0	73.1 ± 10.3	67.2 ± 10.9	**.001**
Female gender	69 (37.3%)	24 (44.4%)	45 (34.4%)	.197
Time after NVAF diagnosis (years)	5.9 ± 6.9	6.4 ± 7.1	5.7 ± 6.9	.483
Type of NVAF				.185
Paroxysmal	64 (34.6%)	14 (25.9%)	50 (38.2%)	
Persistent	36 (19.5%)	14 (25.9%)	22 (16.8%)	
Permanent	85 (45.9%)	26 (48.1%)	59 (45.0%)	
Symptomatic NVAF	118 (63.8%)	33 (61.1%)	85 (64.9%)	.627
History of heart failure	69 (37.3%)	31 (57.4%)	38 (29.0%)	**<.001**
History of coronary artery disease	50 (27.0%)	23 (42.6%)	27 (20.6%)	**.002**
Cardiac implantable electronic device	41 (22.2%)	12 (22.2%)	29 (22.1%)	.990
History of ischemic stroke/TIA	46 (24.9%)	18 (33.3%)	28 (21.4%)	.087
Hypertension	156 (84.3%)	50 (92.6%)	106 (80.9%)	**.047**
Diabetes mellitus	71 (38.4%)	30 (55.6%)	41 (31.3%)	**.002**
History of smoking	72 (38.9%)	21 (38.9%)	51 (38.9%)	.996
Dyslipidemia	127 (68.6%)	42 (77.8%)	85 (64.9%)	.086
Renal replacement therapy	2 (1.1%)	2 (3.7%)	0 (0.0%)	.084
Dementia	4 (2.2%)	2 (3.7%)	2 (1.5%)	.581
History of bleeding	31 (16.8%)	14 (25.9%)	17 (13.0%)	**.032**
CHA_2_DS_2_‐VASc score				**.011**
0	6 (3.2%)	0 (0.0%)	6 (4.6%)	
1	19 (10.3%)	1 (1.9%)	18 (13.7%)	
≥2	160 (86.5%)	53 (98.1%)	107 (81.7%)	
HAS‐BLED score				**<.001**
0	8 (4.3%)	1 (1.9%)	7 (5.3%)	
1–2	128 (69.2%)	25 (46.3%)	103 (78.6%)	
≥3	49 (26.5%)	28 (51.9%)	21 (16.0%)	
Chronic kidney disease	136 (73.5%)	49 (90.7%)	87 (66.4%)	**.001**
Anemia	78 (42.2%)	37 (68.5%)	41 (31.3%)	**<.001**
Left ventricular ejection fraction <50%	28 (17.5%)	13 (28.3%)	15 (13.2%)	**.023**
Antiplatelet	72 (38.9%)	24 (44.4%)	48 (36.6%)	.322
Anticoagulant	138 (74.6%)	41 (75.9%)	97 (74.0%)	.789
Beta blocker	134 (72.4%)	40 (74.1%)	94 (71.8%)	.748
Statin	131 (70.8%)	39 (72.2%)	92 (70.2%)	.786
ACEI/ARB	93 (50.3%)	27 (50.0%)	66 (50.4%)	.962
NT‐proBNP level (pg/ml)	2399.5 ± 6853.0	3,658.3 ± 5201.7	1880.6 ± 7383.2	.109
Soluble ST2 level (ng/ml)	31.3 ± 19.7	39.8 ± 22.3	27.8 ± 17.4	**.001**

*Note*: Data presented as mean ± standard deviation or number and percentage. The bold values are statistically significant *p* < .05.

Abbreviations: ACEI, angiotensin converting enzyme inhibitor; ARB, angiotensin receptor blocker; HF, heart failure; NT‐proBNP level, N‐terminal pro‐brain natriuretic peptide; NVAF, non‐valvular atrial fibrillation; TIA, transient ischemic attack.

### 3.3 Univariate and multivariate analysis

**Figure 2:**
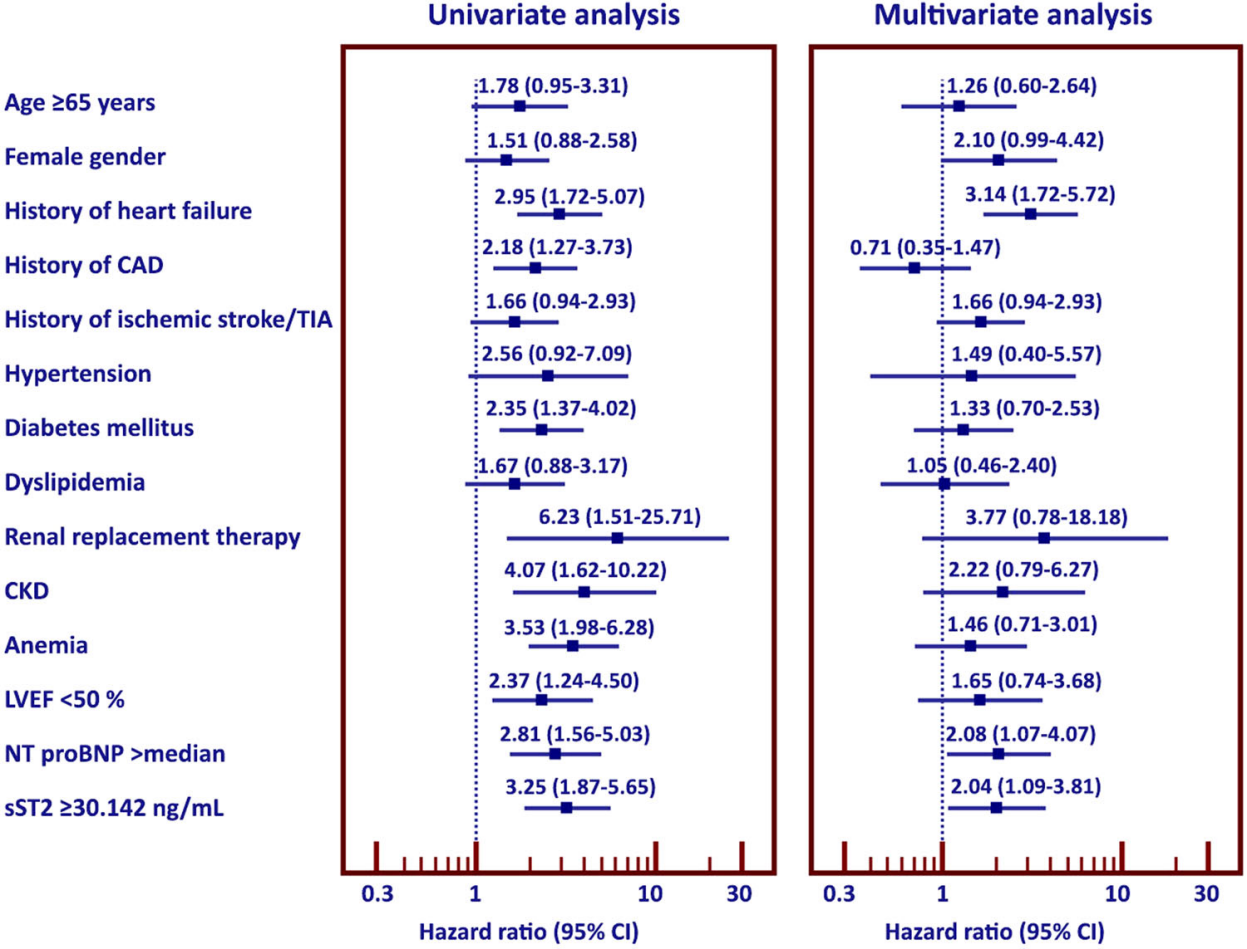
Forest plot of univariate and multivariate analysis for factors that predict heart failure or death. CAD, coronary artery disease; CI, confidence interval; CKD, chronic kidney disease; LVEF, left ventricular ejection fraction; NT‐proBNP level, N‐terminal pro‐brain natriuretic peptide; sST2, soluble ST2; TIA, transient ischemic attack

The results of univariate and multivariate Cox proportional analysis are shown as a forest plot in Figure 2. Factors with *p*‐value <.2 from Table 1 were selected for univariate and multivariate Cox‐proportional Hazard model analysis. Univariate analysis showed history of HF, CAD, DM, RRT, CKD, anemia, LVEF < 50%, NT‐proBNP >median, and sST2 > 30.14 ng/ml to be predictors of HF or death. Subsequent multivariate analysis revealed history of HF, NT‐proBNP >median, and sST2 ≥ 30.14 ng/ml to be independent predictors for HF or death.

### 3.4 Survival analysis

**Figure 3:**
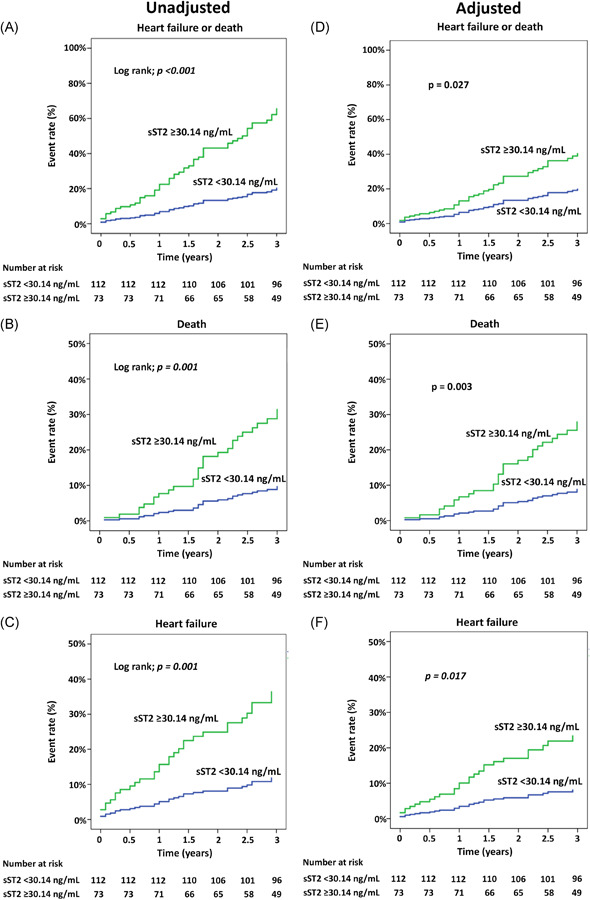
Cumulative rate of heart failure (HF) or death, death, and HF compared between patients with sST2 level ≥30.14 and <30.14 ng/ml. A–C: unadjusted, D–F: adjusted for confounders

The cumulative event rates of HF or death, HF, and death are shown in Figure 3. The event rate in patients with sST2 ≥ 30.14 ng/ml increased as the follow‐up time increased and significantly different from those with sST2 < 30.14 ng/ml both for unadjusted and adjusted model. Moreover, the distance between the two plots (sST2 ≥ 30.14 and sST2 < 30.14 ng/ml) becomes greater as the follow‐up duration increases.

### 3.5 Sensitivity analysis and test of interaction effect

**Figure 4:**
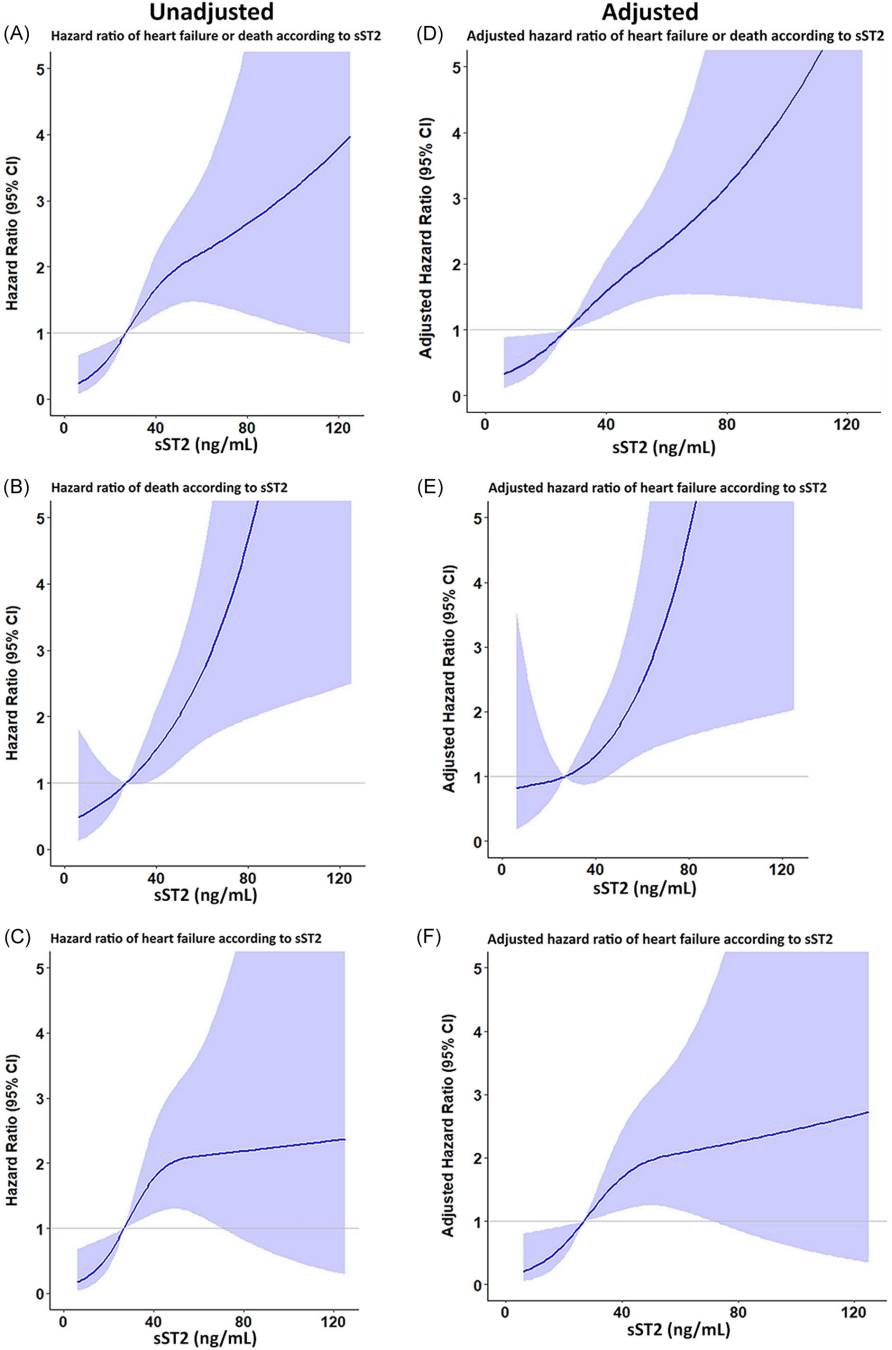
Cubic spline graph showing hazard ratio and 95% confidence interval (CI) heart failure (HF) or death, death, and HF of sST2 as continuous data with A–C: unadjusted, D–F: adjusted for confounders

Sensitivity analysis was performed by treating sST2 as continuous data and testing the effect of sST2 on heart failure or death, death, and heart failure outcome. Restricted cubic spline graph demonstrates that the risk of heart failure or death increased as the levels of sST2 increased both for unadjusted and adjusted model (variables with *p* < .2 from Table 1 were included in the adjusted model) (Figure 4). Figure S1 demonstrates that there were no significant interactions (interaction test *p* > .05) between history of heart failure and sST2 levels (Figure S1A–C) and NT‐proBNP levels and sST2 levels (Figure S1D–F) on each of the clinical outcomes.

Sensitivity analysis was also performed by using median (26.78 ng/ml) of sST2 as a cut‐off and by comparing four groups of sST2 separated by quartiles (1st quartile: <18.54 ng/ml, 2nd quartile: 18.54–26.78 ng/ml, 3rd quartile: 26.78–38.38 ng/ml, 4th quartile: ≥38.38 ng/ml). The results are shown in Figure S2.

Subgroup analysis for the predictive value of sST2 for HF or death showed that sST2 can predict HF or death in patients with AF in the majority of subgroups including age, sex, history of HF, CAD, stroke, hypertension, diabetes, CKD, LVEF, and NT‐proBNP (Figure S3).

## 4 DISCUSSION

This prospective study in patients with nonvalvular AF revealed sST2 to be an independent predictor of death or HF. sST2 was also found to be an independent predictor of HF and death when each of those two study outcomes was considered individually. The importance of sST2 as an independent predictor of outcome was demonstrated in patients with and without history of HF, and in patients with NT‐proBNP ≥median and <median.

Patients with AF had a 3‐fold increased risk of HF, and patients with HF had a 4.5–5.9‐fold increased risk of AF.^14^ Practice guidelines recommend that the treatment of AF focus not only on prevention of ischemic stroke and rate and rhythm control, but also on management of comorbidities, such as HT and DM.^8^ Integrated management of AF patients with oral anticoagulant (OAC) and management of comorbidities have been shown to be associated with better clinical outcomes.^15^

The meta‐analysis global group in chronic heart failure (MAGGIC) risk score has been proposed for the prediction of mortality in patients with chronic HF, including both HF with reduced ejection fraction (HFrEF) and HF with preserved ejection fraction (HFpEF).^16^ Moreover, some biomarkers, such as troponin, BNP, or NT‐proBNP, and sST2, have been shown to improve the performance of models designed to predict the risk of patients with HF.^12^, ^17^ Although we have many data on biomarkers and prognosis of heart failure,^18^, ^19^ there were limited data on the prediction of HF especially in patients with patients with AF. Data from the present study showed history of HF, CKD, and sST2 level ≥30.14 ng/ml (ROC cut off) to be independent predictors of HF in patients with AF. NT‐proBNP level has been recommended not only for the diagnosis, but also for prognostic assessment in patients with HF.^12^, ^17^ BNP can be used to predict risk of HF in high‐risk population.^20^ Natriuretic peptide levels are elevated approximately 20%–30% in patients with AF; therefore, the criteria for diagnosis of HF in patients with AF should be different from those used for diagnosis of HF in patients without AF.^21^ Increased BNP levels predict an increased risk of mortality in patients with and without HF.^22^ Data from the Fushimi AF registry showed that increased BNP levels in patients with AF without known HF were associated with increased risk of mortality, ischemic stroke, and HF.^23^ Data from the same study demonstrated an increased risk of adverse outcome in patients with pre‐existing HF. The results of univariate analysis in our study showed history of HF, CAD, DM, RRT, CKD, anemia, LVEF < 50%, NT‐proBNP >median, and sST2 > 30.14 ng/ml to be predictors of death or HF among patients with AF. Our multivariate analysis revealed history of HF, CKD, and sST2 ≥ 30.14 ng/ml to be independent predictors of HF or death in patients with AF. NT‐proBNP >median was not included in the final multivariate analysis model.

Among patients with HF, a previous study found sST2 level to be stronger than BNP and troponin‐T levels for predicting death and HF in the future.^24^ Among patients with AF, sST2 levels predict recurrence of AF after RF ablation.^25^ In Chinese population, sST2 was shown to be a predictor of HF risk in patients with AF.^26^ Data from European population with anticoagulated AF showed sST2 to be a marker for increased risk of mortality.^27^ The strength of the present study is that we explored both mortality and HF outcome, and both composite and individual outcome. We also performed a separate subanalysis analysis in patients with and without history of HF, and in patients with NT‐proBNP levels ≥median and <median. Our results showed sST2 to be a predictor of HF or death in AF patients regardless of history of HF, and regardless of NT‐proBNP level.

The results of this study suggest several important considerations. First, the risk of HF is high in patients with AF. The rate of HF in AF was even greater than the rate of ischemic stroke/TIA. This finding emphasizes the importance of a management strategy to reduce HF risk. Second, sST2 was shown to be a useful biomarker that can augment clinical data in the prediction of HF. Moreover, the predictive power of sST2 was even greater than that of NT‐proBNP. Third, although we did not have data on sST2‐guided management of HF in patients with AF, previous studies in patients with HF and sinus rhythm showed sST2 level to be significantly associated with reduced HF risk, and that patients with reduced sST2 level after treatment had a better prognosis.^28^, ^29^

### 4.1 Limitations

This study has some mentionable limitations. First, the size of our study population is relatively small, so our study may have been insufficiently powered to identify all statistically significant differences and associations. However, we enrolled all eligible non‐valvular AF patients during our study period. Moreover, the sufficient statistical power of our study may be supported by the fact that we found sST2 ≥ 30.14 ng/ml to be significantly and independently associated with death or HF regardless of history of HF or NT‐proBNP level status. Second, our center is a large tertiary care hospital that is often referred more complex cases, so our results may not be immediately generalizable to AF population seeking/receiving treatment at primary care centers. Third and last, sST2 laboratory data were analyzed only at baseline. sST2 remained a significant predictor for clinical outcomes.

## 5 CONCLUSION

The results of this study revealed sST2 level to be an independent predictor of death or HF in patients with non‐ventricular AF irrespective of history of HF or NT‐proBNP levels.

## CONFLICT OF INTERESTS

All authors declare no personal or professional conflicts of interest, and no financial support from the companies that produce and/or distribute the drugs, devices, or materials described in this report.

## AUTHOR CONTRIBUTIONS

All authors contributed substantially to the following: study conception and design; acquisition or analysis and interpretation of the data; drafting and/or critically revising the article; and, preparing the manuscript for submission to our target journal. All authors are in agreement with both the final version of the manuscript, and the decision to submit this manuscript for journal publication.

## Supporting information

Supporting information.

## Data Availability

The data set that was used to support the results and conclusion of this study is included within the manuscript. Additional data are available from the corresponding author upon reasonable request.
