# Supplementary material for: Soluble ST2 in the prediction of heart failure and death in patients with atrial fibrillation
Source: Clin Cardiol. 2022 Feb 21;45(4):447–56. doi: 10.1002/clc.23799 (PMC9019881; doi:10.1002/clc.23799)

**Supplementary materials**

**Supplementary Table 1.** Rate of clinical outcomes among NVAF patients with sST2 level ≥30.14 ng/ml and <30.14 ng/mL. (A) all patients, (B) History of heart failure, (C) No history of heart failure, (D) NT proBNP ≥median, and (E) NT proBNP <median

| **sST2 groups** | **Number of patients** | **Number of events** | **Per 100**  **person-years** | **Rate per 100 person-years (95% CI)** |  |
| --- | --- | --- | --- | --- | --- |
| 1. **All patients** | | | | | |
| **Heart failure or death** | | | | | |
| sST2 <30.14 | 112 | 20 | 3.139 | 6.37 (3.89-9.84) |  |
| sST2 ≥30.14 | 73 | 34 | 1.883 | 18.06 (12.51-25.23) |  |
| **Death** | | | | | |
| sST2 <30.14 | 112 | 10 | 3.139 | 3.19 (1.53-5.86) |  |
| sST2 ≥30.14 | 73 | 19 | 1.883 | 10.09 (6.08-15.76) |  |
| **Heart failure** | | | | | |
| sST2 <30.14 | 112 | 12 | 3.139 | 3.82 (1.98-6.68) |  |
| sST2 ≥30.14 | 73 | 21 | 1.883 | 11.16 (6.90-17.05) |  |
| 1. **History of heart failure** | | | | | |
| **Heart failure or death** | | | | | |
| sST2 <30.14 | 40 | 11 | 1.074 | 10.24 (5.11-18.33) |  |
| sST2 ≥30.14 | 29 | 20 | 0.734 | 27.24 (16.64-42.08) |  |
| **Death** | | | | | |
| sST2 <30.14 | 40 | 4 | 1.074 | 3.72 (1.01-9.54) |  |
| sST2 ≥30.14 | 29 | 10 | 0.734 | 13.62 (6.53-25.06) |  |
| **Heart failure** | | | | | |
| sST2 <30.14 | 40 | 9 | 1.074 | 8.38 (3.83-15.91) |  |
| sST2 ≥30.14 | 29 | 15 | 0.734 | 20.43 (11.44-33.71) |  |
| 1. **No history of heart failure** | | | | | |
| **Heart failure or death** | | | | | |
| sST2 <30.14 | 72 | 9 | 2.065 | 4.36 (1.99-8.27) |  |
| sST2 ≥30.14 | 44 | 14 | 1.148 | 12.19 (6.67-20.46) |  |
| **Death** | | | | | |
| sST2 <30.14 | 72 | 6 | 2.065 | 2.91 (1.07-6.32) |  |
| sST2 ≥30.14 | 44 | 9 | 1.148 | 7.84 (3.58-14.88) |  |
| **Heart failure** | | | | | |
| sST2 <30.14 | 72 | 3 | 2.065 | 1.45 (0.30-4.25) |  |
| sST2 ≥30.14 | 44 | 6 | 1.148 | 5.23 (1.92-11.38) |  |
| 1. **NT proBNP ≥median** | | | | | |
| **Heart failure or death** | | | | | |
| sST2 <30.14 | 47 | 14 | 1.296 | 10.80 (5.91-18.13) |  |
| sST2 ≥30.14 | 46 | 24 | 1.163 | 20.64 (13.22-30.71) |  |
| **Death** | | | | | |
| sST2 <30.14 | 47 | 7 | 1.296 | 5.40 (2.17-11.13) |  |
| sST2 ≥30.14 | 46 | 14 | 1.163 | 12.04 (6.58-20.20) |  |
| **Heart failure** | | | | | |
| sST2 <30.14 | 47 | 9 | 1.296 | 6.94 (3.18-13.18) |  |
| sST2 ≥30.14 | 46 | 16 | 1.163 | 13.76 (7.86-22.34) |  |
| 1. **NT proBNP <median** | | | | | |
| **Heart failure or death** | | | | | |
| sST2 <30.14 | 65 | 6 | 1.843 | 3.26 (1.19-7.09) |  |
| sST2 ≥30.14 | 27 | 10 | 0.720 | 13.90 (6.66-25.54) |  |
| **Death** | | | | | |
| sST2 <30.14 | 65 | 3 | 1.843 | 1.63 (0.34-4.76) |  |
| sST2 ≥30.14 | 27 | 5 | 0.720 | 6.95 (2.25-16.21) |  |
| **Heart failure** | | | | | |
| sST2 <30.14 | 65 | 3 | 1.843 | 1.63 (0.34-4.76) |  |
| sST2 ≥30.14 | 27 | 5 | 0.720 | 6.95 (2.25-16.21) |  |

**Abbreviations:** sST2, soluble ST2; NT-proBNP level, N-terminal pro-brain natriuretic peptide; CI, confidence interval

**Supplementary Figure 1.** Cubic spline graph showing no evidence of interaction (interaction test p-value >0.05) between A. the presence or absence of history of heart failure and sST2 on heart failure (HF) or death, death, and HF (A-C) and B. NT-proBNP level (≥ or <median) and sST2 on heart failure (HF) or death, death, and HF (D-F). All graphs are displayed as adjusted hazard ratio and 95% confidence interval (CI).

**
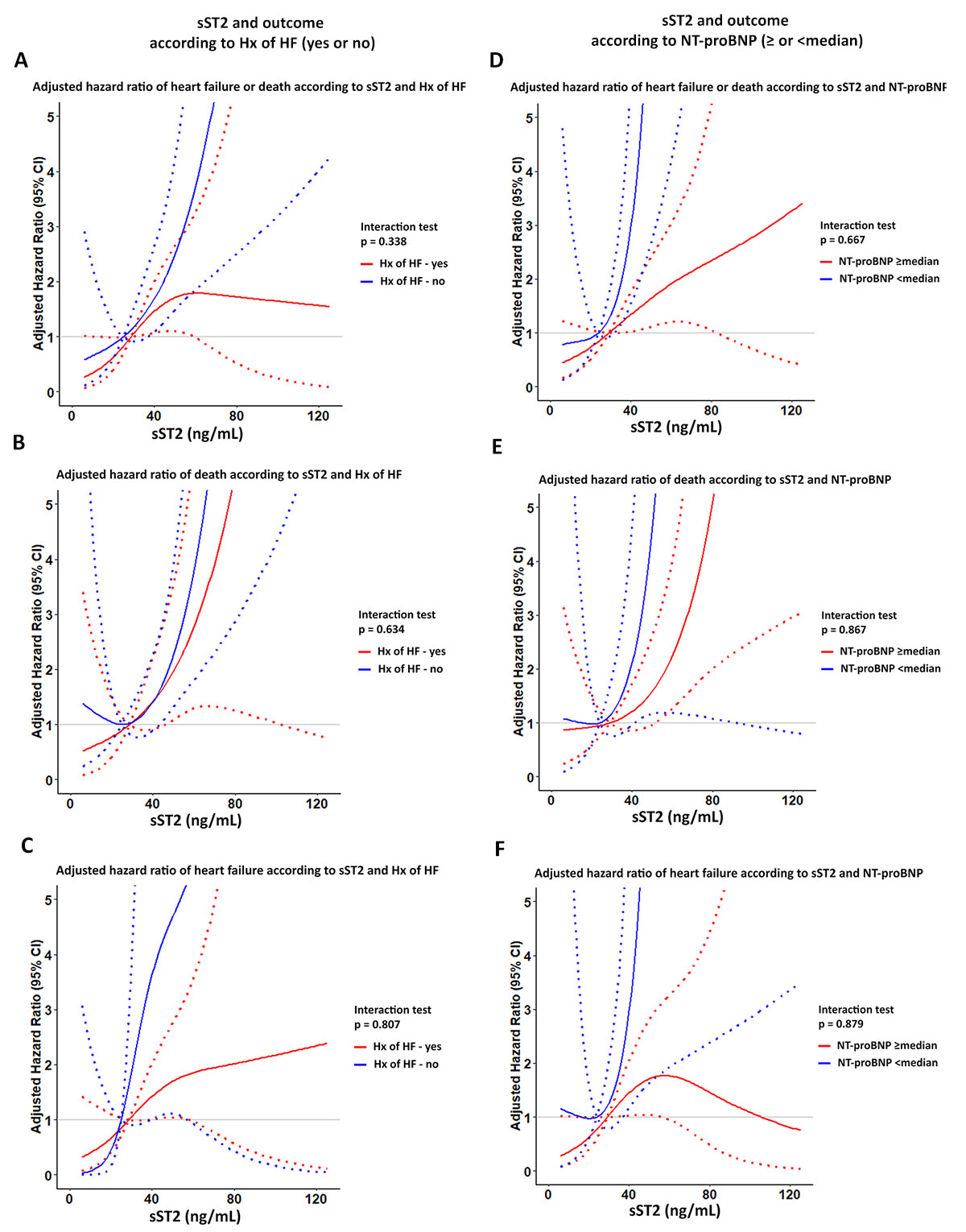
**

**Supplementary Figure 2.** Sensitivity analysis of the difference in the time varying event rate using A-C. sST2 level ≥median and <median (median = 26.78 ng/mL) D-F. sST2 by quartile (1^st^ quartile: <18.54 ng/mL, 2^nd^ quartile: 18.54-26.78 ng/mL, 3rd quartile: 26.78-38.38 ng/mL, 4th quartile: ≥38.38 ng/mL)


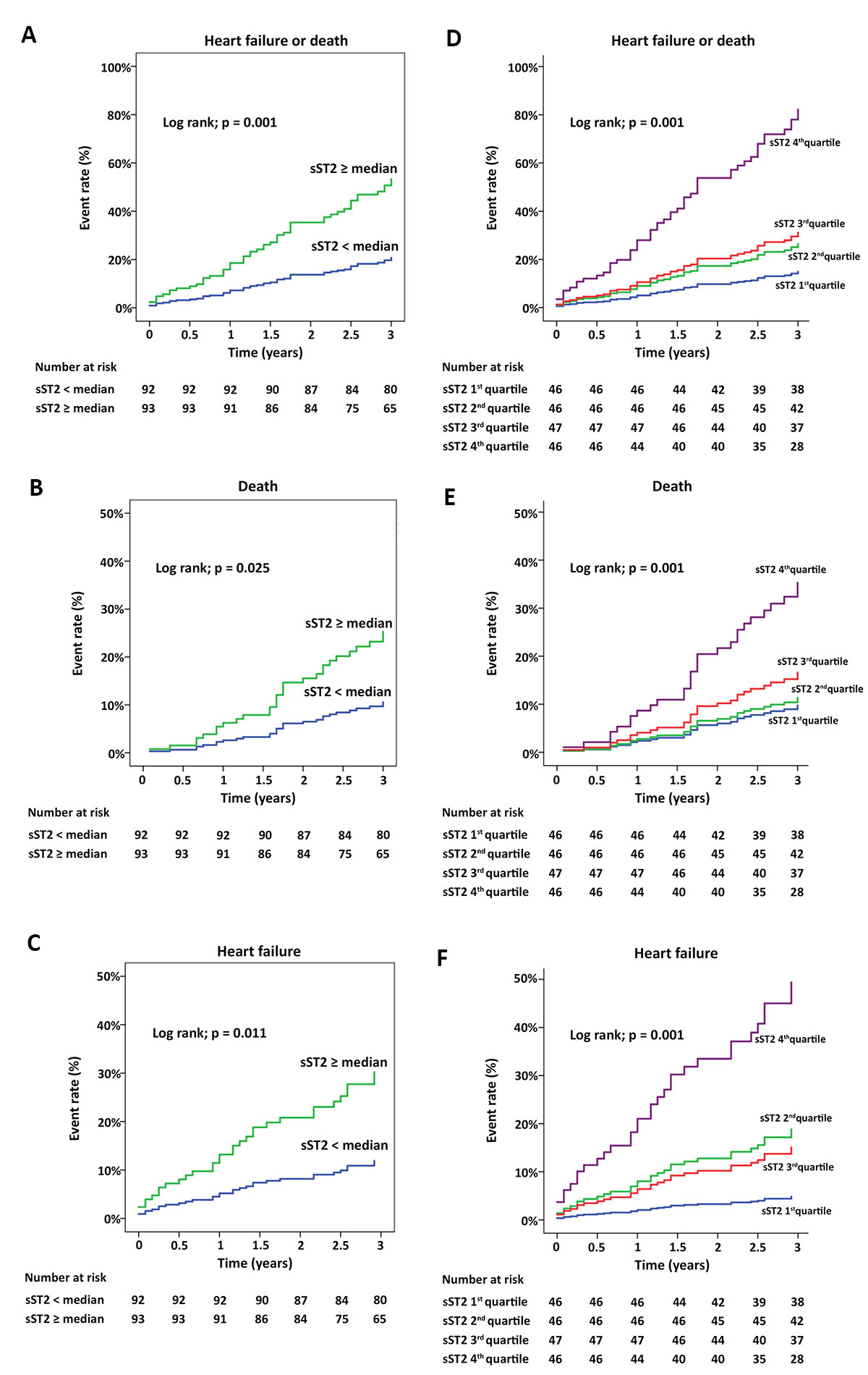


**Supplementary Figure 3**. Subgroup analysis for the predictive value of sST2 for heart failure or death (sST2 = soluble ST2, HR = hazard ratio, CI = confidence interval, NT-proBNP = N-terminal pro- brain natriuretic peptide)


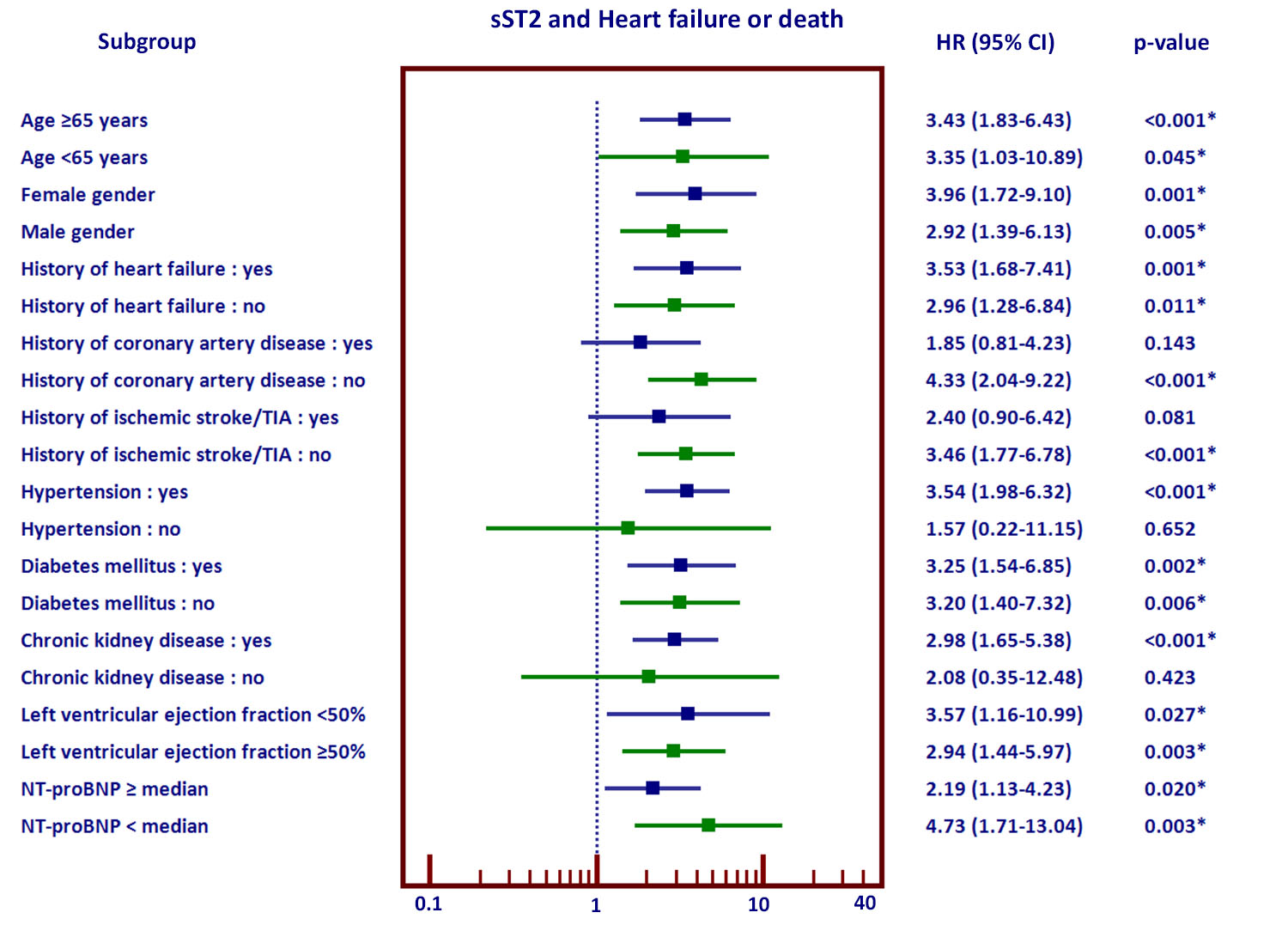

Supplement: Supplementary file 1 — Supporting information. [file CLC-45-447-s001.docx]
